# INK128 Exhibits Synergy with Azoles against *Exophiala* spp. and *Fusarium* spp.

**DOI:** 10.3389/fmicb.2016.01658

**Published:** 2016-10-20

**Authors:** Lujuan Gao, Yi Sun, Chengyan He, Ming Li, Tongxiang Zeng, Qiaoyun Lu

**Affiliations:** ^1^Department of Dermatology, Zhongshan Hospital Fudan UniversityShanghai, China; ^2^Department of Dermatology, Jingzhou Central Hospital, The Second Clinical Medical College, Yangtze UniversityJingzhou, China; ^3^The Second Clinical Medical College, Yangtze UniversityJingzhou, China; ^4^Department of Dermatology, Central Hospital of Xiangyang (Affiliated Hospital of Hubei College of Arts and Sciences)Xiangyang, China

**Keywords:** INK128, TOR inhitibor, synergy, *Fusarium*, *Exophiala*, voriconazole, itraconazole, posaconazole

## Abstract

Infections of *Exophiala* spp. and *Fusarium* spp. are often chronic and recalcitrant. Systemic disseminations, which mostly occur in immunocompromised patients, are often refractory to available antifungal therapies. The conserved target of rapamycin (TOR) orchestrates cell growth and proliferation in response to nutrients and growth factors, which are important for pathogenicity and virulence. INK128 is a second-generation ATP-competitive TOR inhibitor, which binds the TOR catalytic domain and selectively inhibits TOR. In the present study, we investigated the *in vitro* activities of INK128 alone and the interactions of INK128 with conventional antifungal drugs including itraconazole, voriconazole, posaconazole, and amphotericin B against 18 strains of *Exophiala* spp. and 10 strains of *Fusarium* spp. via broth microdilution checkerboard technique system adapted from Clinical and Laboratory Standards Institute broth microdilution method M38-A2. INK128 alone was inactive against all isolates tested. However, favorable synergistic effects between INK128 and voriconazole were observed in 61% *Exophiala* strains and 60% *Fusarium* strains, despite *Fusarium* strains exhibited high MIC values (4–8 μg/ml) against voriconazole. In addition, synergistic effects of INK128/itraconazole were shown in 33% *Exophiala* strains and 30% *Fusarium* strains, while synergy of INK128/posaconazole were observed in 28% *Exophiala* strains and 30% *Fusarium* strains. The effective working ranges of INK128 were 0.125–2 μg/ml and 1–4 μg/ml against *Exophiala* isolates and *Fusarium* isolates, respectively. No synergistic effect was observed when INK128 was combined with amphotericin B. No antagonism was observed in all combinations. In conclusion, INK128 could enhance the *in vitro* antifungal activity of voriconazole, itraconazole and posaconazole against *Exophiala* spp. and *Fusarium* spp., suggesting that azoles, especially voriconazole, combined with TOR kinase inhibitor might provide a potential strategy to the treatment of *Exophiala* and *Fusarium* infections. However, further investigations are warranted to elucidate the underlying mechanism and to determine possible reliable and safe application in clinical practice.

## Introduction

*Exophiala* spp. and *Fusarium* spp. are both increasingly recognized opportunistic pathogen causing cutaneous, subcutaneous and serious invasive infections, especially in immunocompromised and debilitated individuals (Li et al., 2011; Guarro, 2013). Human infection usually occurs as a result of inoculation of the organism through the body surface causing local infection. *E. dermatitidis* is one of the most common cause of chromoblastomycosis (Li et al., 2011), while *Fusarium* spp. causes keratitis and onychomycosis, or locally invasive infections (Guarro, 2013). However, disease in immunocompromised patients often manifests with systemic disseminated fungemia, whose prevalence is effectively growing (Li et al., 2011; Guarro, 2013). *E. dermatitidis* is the leading cause of severe neurotropic phaeohyphomycosis (Li et al., 2011), while fusariosis is, after aspergillosis, the second most common mold infection in humans, among which *F. solani* species complex and *F. oxysporum* species complex are responsible for approximately 80% of the cases (Guarro, 2013).

Prompt antifungal treatment is crucial to prevent life-threatening disease. However, fusariosis is mostly refractory to available treatment, with a high mortality rate for systemic disseminations, which is in accordance with the poor *in vitro* activities of available antifungal drugs against *Fusarium* spp. (Guarro, 2013). In addition, success rate for *Exophiala* spp. infection was only 40–70% despite most antifungal drugs showed favorable *in vitro* activities (Revankar and Sutton, 2010; Kondori et al., 2011; Patel et al., 2013). Optimal treatment remains elusive. Therefore, novel therapeutic strategies are desperately needed.

The target of rapamycin (TOR), which is a conserved serine/threonine kinase in eukaryotes from yeast to humans and orchestrates cell growth and proliferation in response to nutrients and growth factors, is a promising target for the development of novel antifungal strategy (Crespo and Hall, 2002). It has been demonstrated that the TOR pathway regulates cellular responses to nutrients in yeast cells, including proliferation, translation, transcription, autophagy, ribosome biogenesis, lipid homeostasis, morphogenesis and cellular aggregation, which have important implications for pathogenicity and virulence (Crespo and Hall, 2002; Madeira et al., 2015). Therefore, the TOR signaling cascade is an excellent target for the development of broad-spectrum antifungal agents. INK128 is a second-generation ATP-competitive TOR inhibitor, which binds the TOR catalytic domain and selectively inhibits TOR (Hsieh et al., 2012). Previous study revealed synergistic effects between INK128 and itraconazole (ITC), voriconazole (VRC), posaconazole (POS) against *Aspergillus* spp. (Gao et al., 2016). Thus, it is reasonable to speculate that INK128 might also have some antifungal activity and interactions with antifungals against *Exophiala* spp. and *Fusarium* spp.

The aim of this study was to evaluate the effects of INK128 alone and combined with antifungal agents against *Exophiala* spp. and *Fusarium* spp.

## Materials and methods

### Fungal strains

A total of 18 strains of *E. dermatitidis* and 10 strains of *Fusarium* spp. (7 strains of *F. solani*, and 3 strains of *F. oxysporum*) were studied. All strains were clinical isolates. Fungal identification was determined by microscopic morphology and by molecular sequencing of the internal transcribed spacer (ITS) ribosomal DNA (rDNA), as required. *Candida parapsilosis* ATCC 22019 was included to ensure quality control.

### Antifungals and chemical agents

All tested drugs including INK128 (purity ≥ 99%), ITC (purity ≥ 99%), VRC (purity ≥ 99%), POS (purity ≥ 99%), and amphotericin B (AMB; purity ≥ 80%) were purchased in powder form from Selleck Chemicals, Houston, TX, USA and prepared as outlined in the Clinical and Laboratory Standards Institute (CLSI) broth microdilution method M38-A2 (Clinical and Laboratory Standards Institute, 2008). The working concentration ranges of tested drugs were all 0.03–16 μg/ml.

### Inoculum preparation

Conidia harvested from cultures grown for 7 days on Sabouraud dextrose agar (SDA) were suspended in sterile distilled water containing 0.03% Triton and diluted to a concentration of 1–5 × 10^6^ spores/ml, which were than diluted 100 times in RPMI-1640 to achieve a 2-fold suspension more concentrated than the density needed or to approximately 1–5 × 10^4^ spores/ml (Clinical and Laboratory Standards Institute, 2008).

### *In vitro* antifungal activity of individual tested agents

The individual minimal inhibitory concentrations (MICs) of INK128, ITC, VRC, POS and AMB were determined according to M38-A2 method (Clinical and Laboratory Standards Institute, 2008). The 96-well plate was inoculated with 100 μl of the inoculum suspension prepared and 100 μl of the serial diluent of tested drugs. Interpretation of results was performed after incubation at 35°C for 48 h for *Fusarium* spp. and 72 h for *Exophiala* spp., respectively. All tests were performed in triplicate.

### *In vitro* interactions of INK128 and antifungals agents

The interactions between INK128 and antifungal agents against all strains were tested via the microdilution chequerboard technique, adapted from the CLSI M38-A2 microdilution method (Clinical and Laboratory Standards Institute, 2008). As described, a 50 μl of INK128 with serial dilutions were inoculated in horizontal direction and another 50 μl of azoles or AMB with serial dilutions were inoculated in vertical direction on the 96-well plate, which contained 100 μl prepared inoculum suspension. Interpretation of results was performed after incubation at 35°C for 48 h for *Fusarium* spp. and 72 h for *Exophiala* spp., respectively. Drug combination interaction was classified on the basis of the fractional inhibitory concentration index (FICI). The FICI as calculated by the formula: FICI = (Ac/Aa) + (Bc/Ba), where Ac and Bc are the MICs of antifungal drugs in combination, and Aa and Ba are the MICs of antifungal drugs A and B alone (Tobudic et al., 2010). All tests were performed in triplicate.

### Interpretation of results

The MICs were determined as the lowest concentration resulting in complete inhibition of growth (Clinical and Laboratory Standards Institute, 2008). The interaction of INK128 with azoles, or AMB referred to the fractional inhibitory concentration index (FICI), which was classified as follows: FICI of ≤0.5, synergy; FICI of >0.5 to ≤4, no interaction (indifference); FICI of >4, antagonism (Odds, 2003).

## Results

### *In vitro* antifungal activity of individual tested agent

The MIC ranges of individual tested drugs against *E. dermatitidis* isolates were >16 μg/ml for INK128, 0.5–1 μg/ml for ITC, 0.25–0.5 μg/ml for VRC, 0.25–1 μg/ml for POS, and 0.5–1 μg/ml for AMB, respectively (Table 1). The MIC ranges against *Fusarium* spp. are >16 μg/ml for INK128 and ITC, 4–8 μg/ml for VRC and POS, and 2–4 μg/ml for AMB, respectively (Table 2). INK128 individually did not exhibit any significant antifungal activity against all tested strains of *Fusarium* spp. and *Exophiala* spp.

**Table 1:** **MICs and FICIs results with combinations of INK128 with antifungal agents against ***E. dermatitidis*****.

**Strain**		**MIC^a^ (μg/ml)**		**FICI^b^**	**MIC^a^ (μg/ml)**		**FICI^b^**	**MIC^a^ (μg/ml)**		**FICIb**	**MIC^a^ (μg/ml)**		**FICI^b^**
	**INK128**	**ITC**	**INK128/ITC**		**VRC**	**INK128/VRC**		**POS**	**INK128/POS**		**AMB**	**INK128/AMB**	
*E. dermatitidis* (1)	>16	0.5	0.125/0.25	I	0.25	0.125/0.03	S	0.5	1/0.25	I	1	0.5/1	I
*E. dermatitidis* (2)	>16	1	0.5/0.5	I	0.25	0.125/0.06	S	1	0.25/0.125	S	1	0.5/1	I
*E. dermatitidis* (3)	>16	1	2/0.25	S	0.25	0.25/0.03	S	0.5	0.125/0.5	I	1	0.5/0.5	I
*E. dermatitidis* (4)	>16	0.5	0.25/0.125	S	0.25	0.25/0.06	S	0.25	0.125/0.25	I	1	0.25/1	I
*E. dermatitidis* (5)	>16	0.5	0.5/0.06	S	0.25	0.25/0.03	S	0.5	0.125/0.125	S	1	0.5/1	I
*E. dermatitidis* (6)	>16	0.5	0.25/0.25	I	0.25	0.125/0.06	S	0.5	0.125/0.5	I	0.5	0.25/0.5	I
*E.dermatitidis* (7)	>16	0.5	0.125/0.25	I	0.5	0.125/0.25	I	0.5	0.125/0.5	I	1	1/0.5	I
*E.dermatitidis* (8)	>16	0.5	0.5/0.125	S	0.25	0.25/0.06	S	0.25	0.125/0.125	I	0.5	1/0.5	I
*E.dermatitidis* (9)	>16	0.5	0.125/0.5	I	0.25	0.125/0.25	I	0.5	0.25/0.5	I	1	0.5/1	I
*E.dermatitidis* (10)	>16	1	0.125/1	I	0.25	0.125/0.25	I	0.5	0.125/0.5	I	0.5	0.5/0.25	I
*E. dermatitidis* (11)	>16	1	0.5/1	I	0.25	0.25/0.125	I	0.5	0.125/0.5	I	1	1/0.5	I
*E. dermatitidis* (12)	>16	0.5	0.125/0.5	I	0.25	0.25/0.03	S	0.5	0.125/0.125	S	1	0.5/1	I
*E. dermatitidis* (13)	>16	0.5	2/0.5	I	0.5	2/0.125	S	0.5	0.25/0.125	S	1	0.5/1	I
*E. dermatitidis* (14)	>16	1	0.5/0.25	S	0.5	0.25/0.25	I	0.5	0.125/0.5	I	0.5	0.25/0.5	I
*E. dermatitidis* (15)	>16	1	0.25/0.5	I	0.5	0.25/0.125	S	0.5	0.125/0.5	I	1	1/0.5	I
*E. dermatitidis* (16)	>16	1	0.125/0.5	I	0.5	0.5/0.25	I	0.5	0.125/0.5	I	0.5	0.25/0.25	I
*E. dermatitidis* (17)	>16	0.5	0.5/0.25	I	0.25	0.25/0.25	I	0.5	0.25/0.25	I	1	0.5/1	I
*E. dermatitidis* (18)	>16	1	0.25/0.25	S	0.25	0.125/0.03	S	0.5	0.25/0.125	S	1	0.5/1	I

a *The MIC is the concentration achieving 100% growth inhibition*.

b *S, synergy (FICI of ≤0.5); I, no interaction (indifference) (0.5 < FICI ≤4)*.

**Table 2:** **MICs and FICIs results with combinations of INK128 with antifungal agents against ***Fusarium*** spp**.

**Strain**		**MIC^a^ (μg/ml)**		**FICI^b^**	**MIC^a^ (μg/ml)**		**FICI^b^**	**MIC^a^ (μg/ml)**		**FICI^b^**	**MIC^a^ (μg/ml)**		**FICI^b^**
	**INK128**	**ITC**	**INK128/ITC**		**VRC**	**INK128/VRC**		**POS**	**INK128/POS**		**AMB**	**INK128/AMB**	
*F. solani* (1)	>16	>16	4/8	I	8	2/2	S	4	2/4	I	4	8/4	I
*F. solani* (2)	>16	>16	4/8	I	4	2/2	I	4	2/2	I	2	8/2	I
*F. solani* (3)	>16	>16	4/4	S	8	2/2	S	4	4/2	I	4	8/4	I
*F. solani* (4)	>16	>16	4/8	I	8	1/2	S	4	8/4	I	4	8/4	I
*F. solani* (5)	>16	>16	4/16	I	8	2/4	I	4	2/1	S	2	4/2	I
*F. solani* (6)	>16	>16	8/16	I	8	2/2	S	4	2/4	I	4	4/2	I
*F. solani* (7)	>16	>16	4/4	S	8	2/1	S	8	2/2	S	2	8/2	I
*F. oxysporum* (1)	>16	>16	8/16	I	8	2/4	I	4	4/4	I	4	4/4	I
*F. oxysporum* (2)	>16	>16	8/16	I	8	2/4	I	8	2/2	S	4	4/4	I
*F. oxysporum* (3)	>16	>16	4/4	S	8	2/2	S	4	8/4	I	2	4/1	I

a *The MIC is the concentration achieving 100% growth inhibition*.

b *S, synergy (FICI of ≤0.5); I, no interaction (indifference) (0.5 < FICI ≤4)*.

### *In vitro* interactions between INK128 and antifungal agents

When INK128 was combined with VRC, the MICs of INK128 and VRC against *Exophiala* spp. decreased to 0.125–2 μg/ml and 0.03–0.25 μg/ml, respectively, demonstrating favorable synergistic effects against 11 (61%) strains of *E. dermatitidis* (Table 1). Similarly, this INK128/VRC combination also showed favorable synergism against 6 (60%) strains of *Fusarium* isolates (Table 2), where the MIC ranges of INK128 and VRC decreased to 1–2 μg/ml and 1–4 μg/ml, respectively. The effective working ranges of INK128 in this combination were 0.125–2 μg/ml and 1-2 μg/ml against *Exophiala* spp. and *Fusarium* spp., respectively.

When INK128 was combined with POS, the MIC ranges of INK128 and POS against *Exophiala* spp. decreased to 0.125–1 μg/ml and 0.125–0.5 μg/ml, respectively (Table 1). The MIC ranges of INK128 and POS against *Fusarium* spp. decreased to 2–8 μg/ml and 1–4 μg/ml, respectively (Table 2). The INK128/POS combination revealed synergistic effects against only 5 (28%) strains of *Exophiala* spp. and 3(30%) strains of *Fusarium* spp. (Tables 1, 2). The effective working ranges of INK128 in INK128/POS combination were 0.125–0.25 μg/ml and 2 μg/ml against *Exophiala* spp. and *Fusarium* spp., respectively.

When INK128 was combined with ITC, the MIC ranges of INK128 and ITC against *Exophiala* spp. decreased to 0.125–2 μg/ml and 0.06–1 μg/ml, respectively. The MIC ranges of INK128 and ITC against *Fusarium* spp. decreased to 4–8 μg/ml and 4–16 μg/ml, respectively. Synergistic effects of the INK128/ITC combination were only observed in only 6 (33%) strains of *Exophiala* spp. and 3(30%) strains of *Fusarium* spp. (Tables 1, 2). The effective working ranges of INK128 in INK128/ITC combination were 0.25–2 μg/ml and 4 μg/ml against *Exophiala* spp. and *Fusarium spp*., respectively.

No synergistic effect was observed when INK128 was combined with AMB. No antagonism was observed in these combinations.

## Discussion

TOR kinase is the central element of TOR signaling pathway, which has been widely investigated for years since its discovery and has been recognized as a central controller of cell growth in eukaryotes (Crespo and Hall, 2002). The pharmaceutical potential of rapamycin, the classical allosteric inhibitor of TOR, was originally discovered in a screen for novel antifungal agents. Rapamycin was found to exhibit potent antifungal effects against a variety of species including *Candida* spp., *Cryptococcus* spp., *Aspergillus* spp., *Fusarium* spp., *Penicillium* spp., and dermtophytes (Rohde and Cardenas, 2004). However, rapamycin failed to be used as an antifungal due to its even more potent immunosuppressive property.

INK128, a highly potent, orally active TOR kinase inhibitor, was originally developed for cancer treatment (Hsieh et al., 2012). It has been demonstrated that oral administration of INK128 in mice has high absorption and bioavailability, with doses of 3 mg/kg giving a Cmax of 0.599 μg/ml in plasma (Hsieh et al., 2012). Moreover, previous study has shown that INK128 did not inhibit *in vitro* peripheral blood lymphocytes proliferation at concentrations of up to 1 μM (0.31 μg/ml), and daily administration of INK128 at a dose up to 5 mg/kg in humanized mice over a 2-week period showed no obvious toxicity (Heredia et al., 2015), suggesting that INK128 is not as immunosuppressive as rapamycin.

Previously, we have demonstrated that INK128 exhibited synergistic effects with ITC (65%), VRC (61%), POS (50%) against *Aspergillus* spp. (Gao et al., 2016). In the present study, we investigated the *in vitro* antifungal activity of INK128 alone and combined with antifungal agents against *Exophiala* spp. and *Fusarium* spp. The results revealed that INK128 alone was inactive against all strains tested, as was demonstrated against *Aspergillus* isolates (Gao et al., 2016). Nevertheless, synergistic activities between INK128 and VRC (61%), ITC (33%), POS (28%) were observed in *Exophiala* spp. Although, the MIC values of ITC, VRC and POS against *Fusarium* strains were much higher that those against *Exophiala* spp., synergistic effects between INK128 and VRC (60%), ITC(30%), POS (30%) against *Fusarium* isolates were comparable to those of *Exophiala* spp. The effective working ranges of INK128 were 0.125–2 μg/ml and 1–4 μg/ml against *Exophiala* spp. and *Fusarium* spp., respectively. No interaction between INK128 and AMB was observed. Moreover, no antagonism was observed.

Synergistic effects between INK128 and ITC or POS against *Exophiala* spp. and *Fusarium* spp. were less frequent than those observed in *Aspergillus* spp. (Gao et al., 2016). However, synergy between INK128 and VRC was comparable among *Exophiala* spp., *Fusarium* spp., and previously tested *Aspergillus* spp. (Gao et al., 2016), despite *Fusarium* spp. showed relatively poor antifungal susceptibility profile. It is important to note that even with high MICs against VRC (4–8 μg/ml), synergy between INK128 and VRC was observed in up to 60% *Fusarium* strains, implicating that INK128 could enhance the *in vitro* susceptibility of VRC-inactive *Fusarium* strains. The effective working ranges of INK128 against *Exophiala* spp. were mostly within 0.125–0.25 μg/ml, which could be achieved safely as mentioned above. Compared to *Exophiala* spp., the working ranges of INK128 against *Fusarium* spp. were higher, which might associated with the inherently high MICs of *Fusarium* spp. to most available antifungal medications.

Previous studies have demonstrated synergistic interactions between rapamycin and AMB (70%), ITC (50%), POS (40%) against *Mucorales* (previously referred to as *zygomycetes*) through broth microdilution checkerboard procedure (Dannaoui et al., 2009). Futhermore, antagonism of rapamycin/ITC, and indifference to significant antagonism of rapamycin/POS against *Mucorales* (previously referred to as *zygomycetes*) have also been reported (Dannaoui et al., 2009; Narreddy et al., 2010). However, there was no interaction between INK128 and AMB against tested *Exophiala* spp. and *Fusarium* spp. in the present study, or *Aspergillus* spp. in the previous study (Gao et al., 2016). No antagonism between INK128 and POS or ITC was observed. The explanation of the difference between INK128 and rapamycin might lie in the different mechanism through which INK128 and rapamycin interact with TOR and antifungals, and different response of tesed species against these antifungal drugs.

In summary, the present study extends previous findings in the combination interactions between TOR inhibitors, especially INK128, and conventional antifungals. INK128 could enhance the *in vitro* antifungal activity of VRC, ITC, and POS against *Exophiala* spp. and *Fusarium* spp. More importantly, the inexistence of immunosuppression of INK128 suggests that the combination of azoles with INK128 might provide a safe alternative strategy to the treatment of *Exophiala* and *Fusarium* infections. However, further investigations are warranted to elucidate the underlying mechanism and to determine possible reliable and safe application in clinical practice.

## Author contributions

LG and YS: Conceived and designed the study; CH and YS: Performed all the experiments; LG and QL: Analyzed the data and wrote the manuscript; ML and TZ: Provided general guidance and revised the manuscript.

## Funding

This work was supported by grants 31400131 (LG) and 81401677 (YS) from National Natural Science Foundation of China, grant 2015ZSYXQN21 from Outstanding Youth Project of Zhongshan Hospital Fudan University (LG) and grant WJ2015MB281 from Hubei Province Health and Family Planning Scientific Research Project (YS).

### Conflict of interest statement

The authors declare that the research was conducted in the absence of any commercial or financial relationships that could be construed as a potential conflict of interest.
